# Identification of a set of endogenous reference genes for miRNA expression studies in Parkinson’s disease blood samples

**DOI:** 10.1186/1756-0500-7-715

**Published:** 2014-10-10

**Authors:** Alice Serafin, Luisa Foco, Hagen Blankenburg, Anne Picard, Stefano Zanigni, Alessandra Zanon, Peter P Pramstaller, Andrew A Hicks, Christine Schwienbacher

**Affiliations:** Center for Biomedicine, European Academy Bozen/Bolzano (EURAC), 39100 Bolzano, Italy; Department of Neurology, General Central Hospital, 39100 Bolzano, Italy; Department of Neurology, University of Lübeck, Lübeck, 23538 Germany; Affiliated Institute of the University of Lübeck, Lübeck, Germany

**Keywords:** qRT-PCR, snoRNA, snRNA, geNorm algorithm, Normfinder algorithm, Comparative delta-Ct

## Abstract

**Background:**

Research on microRNAs (miRNAs) is becoming an increasingly attractive field, as these small RNA molecules are involved in several physiological functions and diseases. To date, only few studies have assessed the expression of blood miRNAs related to Parkinson’s disease (PD) using microarray and quantitative real-time PCR (qRT-PCR). Measuring miRNA expression involves normalization of qRT-PCR data using endogenous reference genes for calibration, but their choice remains a delicate problem with serious impact on the resulting expression levels. The aim of the present study was to evaluate the suitability of a set of commonly used small RNAs as normalizers and to identify which of these miRNAs might be considered reliable reference genes in qRT-PCR expression analyses on PD blood samples.

**Results:**

Commonly used reference genes snoRNA RNU24, snRNA RNU6B, snoRNA Z30 and miR-103a-3p were selected from the literature. We then analyzed the effect of using these genes as reference, alone or in any possible combination, on the measured expression levels of the target genes miR-30b-5p and miR-29a-3p, which have been previously reported to be deregulated in PD blood samples.

**Conclusions:**

We identified RNU24 and Z30 as a reliable and stable pair of reference genes in PD blood samples.

**Electronic supplementary material:**

The online version of this article (doi:10.1186/1756-0500-7-715) contains supplementary material, which is available to authorized users.

## Background

MicroRNAs (miRNAs) are small non-coding RNAs of 20–22 nucleotides involved in transcriptional and post-transcriptional regulation of gene expression. MiRNAs function via base-pairing with complementary sequences within target mRNA molecules, usually resulting in gene silencing via translational repression or target degradation [1]. MiRNAs are involved in several physiological functions, such as cell cycle, apoptosis, proliferation, differentiation and development [2]. In recent years the research on miRNAs has increased in intensity because of their involvement not only in physiological processes but also in different diseases. Several studies have shown abnormal expression levels of miRNAs in different pathologies, including Parkinson’s disease [3]. The main clinical hallmarks of this neurodegenerative disorder are resting tremor, muscular rigidity, bradykinesia, impaired balance and coordination. Pathologically PD is characterized by the loss of the dopaminergic neurons in the motor control region substantia nigra and an accumulation of protein-filled structures called Lewy bodies [4]. Lewy bodies and other brain specific diagnostic signs cannot be observed until after death of the PD affected individuals. Biomarkers from easy accessible resources, such as peripheral blood, plasma, serum, urine, saliva, and eventually cerebrospinal fluid could be used to detect and monitor the disease much earlier, even before symptoms appear. Unfortunately to date no proven biomarkers are available for the diagnosis of PD [5]. MiRNAs are strong and specific gene regulators and therefore promising candidates to be diagnostic markers and therapeutic targets. Peripheral blood can be considered as a potential diagnostic tool because it is readily obtainable and reflects dynamically a system-wide biology [6].

To date, only few studies have assessed the expression of blood miRNAs related to Parkinson’s disease using microarray and quantitative real-time PCR [7, 8]. Several methods have been developed to measure miRNA expression [9]. Of these methods qRT-PCR is superior due to its sensitivity, specificity and linear dynamic range of quantification [10, 11] and is therefore generally accepted as the gold standard for accurate gene quantification and small RNA profiling. To distinguish true biological changes from technical variations, several variables must be considered and controlled for gene-expression analysis, such as the amount of the starting material and differences between tissues in overall transcriptional activity. The use of multiple stable reference genes is generally necessary as the method of choice for qRT-PCR data normalization. The normalization procedure is adapted to compare the expression levels of target genes with the expression of a reference gene to obtain a relative quantification of the investigated target gene [12]. The expression of an ideal reference gene should not vary in the tissues or cells under investigation and in response to treatment or presence of a pathology [13]. Accurate expression profiling is critically dependent on technical variations due to the applied normalization strategies. A misleading estimation of the expression analysis data can arise if only a single or improper reference genes are used [14]. These stable reference genes can be identified from a set of candidate reference genes in a pilot experiment on a selection of samples that are representative for the experimental conditions under investigation. Different algorithms, such as geNorm and Normfinder allow the ranking of candidate reference genes according to their stability and indicate the optimal number and combination of reference genes required for accurate normalization of gene expression [10]. In the case of miRNA expression profiling, only few candidate reference miRNAs, including miRNA 103, have been identified [15, 16]. Typically, other small endogenous noncoding RNAs such as small nuclear (snRNAs) and small nucleolar RNAs (snoRNAs) have been used [10] as they share similar properties, such as RNA stability and size and are expressed abundantly. Unfortunately their expression is not always stable [17]. As no universal reference gene has so far been identified, there is no substitute for empirical validation of normalization that is appropriate to the particular experimental design and goals [16]. Recently expression studies of circulating miRNAs in serum or plasma as potential biomarkers for PD are emerging [18, 19]. In these studies synthetic, non-human spike-in miRNAs, used frequently to monitor RNA purification and retrotranscription efficiencies, are almost exclusively used as well for normalization in expression analyses, because of the lack of established reference genes [9]. Moreover, new emerging technologies including Droplet Digital PCR technology, which provides an absolute quantification of nucleic acids, are trying to overcome the problem of the normalization, but for now they are available only in few laboratories.

The aim of the present study was to evaluate the suitability of a set of commonly used small RNAs as normalizers and to identify which of these miRNAs might be considered reliable reference genes in qRT-PCR expression analyses on PD blood samples. We compared the potential reference genes, snoRNA RNU24 (RNU24), snRNA RNU6B (RNU6B), snoRNA Z30 (Z30) and miR-103a-3p to find the best set of reference genes to use for normalization using (i) comparative delta-Ct [20], (ii) the NormFinder algorithm [21] and (iii) qbasePLUS, an improved version of the geNorm algorithm [14]. The effect of different reference genes used on the relative quantification was shown using the target genes miR-30b-5p and miR-29a-3p, previously shown to be differentially expressed in PD blood samples [7, 8].

## Methods

### Patient and control samples collection

38 patients affected by idiopathic Parkinson’s disease (PD) attending the Movement Disorders outpatient clinic of the Bolzano Hospital (Italy) were enrolled. Diagnosis of PD was made according to Gelb PD criteria [22]. All the patients were treated with Levodopa. The subjects with cognitive impairment or unable to sign informed consent or affected by atypical Parkinsonism were excluded. 38 disease-free controls matched for sex and age (range ± 3 years), collected among spouses or unrelated companions of the patients, were also enrolled. The study was approved by the ethics committee of the Bolzano Hospital and all of the enrolled subjects provided written informed consent to participate.

### RNA isolation and quality control

Whole peripheral blood from patients and controls was collected per participant in a Na_3_-Citrate buffered Venosafe® Plastic Tube (VF-054SBCS07, Terumo) and stored at room temperature. Within 6 hours of blood collection the peripheral blood mononuclear cells (PBMCs) were isolated after lysis of red blood cells by Red Blood Cell (RBC) Lysing Buffer. Total RNA, including small RNAs, was extracted from white blood cells using TRIzol® reagent (Invitrogen) according to the manufacturer’s instructions and stored at -80°C.

The quality and quantity of the extracted RNA was assessed by agarose gel electrophoresis and by Experion™ Automated Electrophoresis System (Bio-Rad Laboratories s.r.l., Milano, Italy), using Experion™ StdSens Analysis Kit (Bio-Rad Laboratories s.r.l., Milano, Italy). The RNA Quality Indicator (RQI) feature of Experion software allows estimating the level of the integrity of eukaryotic total RNA samples. All 76 collected samples showed an a RQI ≥ 8.0 and were included in the expression analyses.

### Reverse Transcription and gene specific quantitative real time PCR

Expression analyses were performed by single gene specific stem loop RT-PCR. Reverse transcription (RT) reactions were performed on 7 ng of total RNA using the TaqMan miRNA Reverse Transcription Kit (Applied Biosystem; Part.No 4366597) and miRNA-specific stem-loop primers (Table 1) in a scaled down volume of 10 μl RT reaction, according to the manufacturer’s instructions.

**Table 1:** **TaqMan**® **MicroRNA Assays and relative amplification efficiencies**

Gene name	mirBase accession/NCBI ref sequence	Assay ID	Sequence/Mature miRNA sequence	PCR amplification efficiency	Correlation coefficient (r ^2^)
hsa-miR-30b-5p	MIMAT0000420	602	UGUAAACAUCCUACACUCAGCU	97.50%	0.983
hsa-miR-29a-3p	MIMAT0000086	2112	UAGCACCAUCUGAAAUCGGUUA	97.40%	0.989
hsa-miR-103a-3p	MIMAT0000101	439	AGCAGCAUUGUACAGGGCUAUGA	96.90%	0.981
RNU24	NR_002447	1001	AUUUGCUAUCUGAGAGAUGGUGAUGACAUUUUAAACCACCAAGAUCGCUGAUGCA	90.40%	0.917
Z30	AJ007733	1092	UGGUAUUGCCAUUGCUUCACUGUUGGCUUUGACCAGGGUAUGAUCUCUUAAUCUUCUCUCUGAGCUG	97.10%	0.979
RNU6B	NR_002752	1093	CGCAAGGAUGACACGCAAAUUCGUGAAGCGUUCCAUAUUUUU	79.90%	0.967

Gene name and relative TaqMan® MicroRNA Assays, sequence, standard curve PCR amplification efficiency and r^2^, the standard curve correlation coefficient.

The thermal cycling parameters of reverse transcription were 30 min at 16°C, 30 min at 42°C, and 5 min at 85°C. The cDNA samples were diluted in nuclease-free water and stored at -20°C.

All quantitative real-time PCR reactions were performed in triplicate on Bio-Rad 96CFX instrument (Bio-Rad Laboratories s.r.l., Milano, Italy), in scaled down 10 μl reaction volumes using 0.7 μl of RT product per reaction using Universal MasterMixII, no UNG and gene specific TaqMan® small RNA Assays (Applied Biosystem®; Table 1) according to the manufacturer’s instructions. All reactions were performed on hard shell PCR plates (Bio-Rad Laboratories Inc. Part.No HSP9645), sealed using adhesive Microseal ‘B’ Films (Bio-Rad Laboratories Inc. Part.No RSN 102595). One reference sample and one NTC (blank) were included in triplicate on each plate. The thermal cycling parameters were 10 min at 95°C followed by 40 cycles of 15 sec at 95°C and 1 min at 60°C.

PCR amplification efficiencies for all target and reference genes were determined from the slope of the log-linear portion of calibration curves, generated from a two-fold dilution series of one reference sample at five dilution points for three technical replicates for each gene assay (Table 1). PCR reactions were performed as described previously for the quantitative real-time PCR reactions. The relative expression of miR-30b-5p and miR-29a-3p were assessed using the Bio-Rad CFX Manager v1.6 (Bio-Rad Laboratories s.r.l., Milano, Italy) software. For normalization, different combinations of reference genes among RNU24, RNU6B, Z30 and miR-103a-3p were used.

### Statistical analysis

In order to determine the expression stability of the four candidate reference genes, statistical analyses of their expression across all samples were performed using three different algorithms: NormFinder [21], GeNorm [14], and comparative delta-Ct method [20]. NormFinder computes a stability measure and the lowest value indicates the most stable gene expression. Samples are grouped to allow direct estimation of expression variation, ranking genes according to the similarity of their expression profiles by using a model-based approach. NormFinder takes into account inter- and intragroup variation for normalization factor calculations avoiding misinterpretation caused by artificial selection of co-regulated genes. GeNorm computes an M value describing the variation of a gene compared to all other candidate genes. Similarly to the NormFinder stability value, lower M values indicate stable gene expression. Specifically, stable reference genes are supposed to have an M value smaller than 1.5.

Comparative delta-Ct compares relative expression of pairs of genes within each sample set and ranks stability of reference genes according to the repeatability of the gene expression difference.

Scatter plots and box plots were used to visualize the presence of outliers in the expression Ct values; outlier values, as evidenced by the box plot, were labelled for possible subsequent removal. Medians and interquartile ranges were produced to summarize data. The medians were calculated on the differences of the expression between each case and its matched control.

For miR-29a-3p and miR-30b-5p, the distribution of the difference of the expression levels between cases and controls, within each matched pair and normalized with different combinations of reference genes, was checked using a Skewness-Kurtosis test for normality. A subset of the ladder of powers for variable transformation in case of departure from normality was evaluated [23].

Relative expression of miR-29a-3p and miR-30b-5p was analysed using a Wilcoxon matched-pairs signed-ranks test. Although 15 tests for each microRNA were performed, no adjustment for multiple comparisons was done. This is because in practice the only test that would have been performed is the one based on data *a priori* normalized for the best reference gene combination. Spearman’s rank correlation coefficients were computed on Ct reference gene data and on normalized miR-29a-3p and miR-30b-5p data. GeNorm analysis was performed using Biogazelle’s qbasePLUS software (Bio-Rad Laboratories Inc.) and NormFinder analysis was performed using “NormFinder.xla”, a Microsoft Excel-based Visual Basic application. All other analyses were performed using Stata 12 [24].

## Results

The selection of the best set of endogenous reference genes for gene expression studies in Parkinson’s disease blood samples was based on the efficiency of the TaqMan® MicroRNA Assays, the quality of the related expression data, and on the expression stability analysis.

### Evaluation of the amplification efficiencies of the TaqMan® MicroRNA Assays

The hallmarks for an accurate and optimized qRT-PCR assay are a linear correlation coefficient (r^2^) equal or greater than 0.98 and a PCR amplification efficiency from 90% to 110% [11]. The r^2^ value indicates the quality of the fit of the standard curve to the plotted data points. Primer efficiency indicates the amplicon doubling rate of a specific primer pair during a PCR. An efficiency of 100% indicates that the cDNA target is duplicated at every PCR cycle during the exponential phase. The efficiencies of the TaqMan® MicroRNA Assays of the four candidate reference genes (RNU24, RNU6B, Z30 and miR-103a-3p), and the two target genes (miR-30b-5p and miR-29a-3p) were calculated from the slope of the log-linear portion of calibration curves as described (Materials and Methods; Reverse Transcription and quantitative real time PCR) and are reported in Table 1. RNU24, Z30, miR-103a-3p, miR-30b-5p, and miR-29a-3p showed amplification efficiencies and r^2^ values ranging respectively between 90.4% and 97.5% and between 0.92 and 0.99 demonstrating a high performance. In contrast, RNU6B showed a low amplification efficiency of 79.9% with a correlation coefficient of 0.967.

### Data quality control

**Figure 1:**
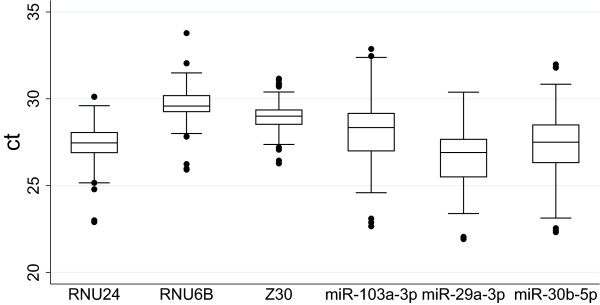
**Expression levels in analysed genes.** Box plot of raw Ct values to inspect the data. y axis: Ct values; x axis: miRNAs analyzed. Boxes: interquartile range, central line is the median; Whiskers: upper and lower adjacent values; Dots: outside values.

The analysis of the generated Ct data revealed the presence of a few outliers, as evidenced in Figure 1. Because no evident technical reason could be found to exclude them, given the high quality of the extracted RNA as evaluated by Experion RNA chip electrophoresis, and the overall high performance of the qRT-PCR reactions, they were not removed from the analyses, but they were rather considered an expression of normal biological variability. The differences of the relative gene expression in cases and in controls, within each matched pair, did not always follow a normal distribution. Since no unique transformation was able to restore deviations from normality, a non-parametric Wilcoxon matched-pairs signed-ranks test was subsequently used to analyse data.

### Expression stability analysis

The results of the expression stability assessment of Z30, RNU24, RNU6B and miR-103a-3p genes among all analysed samples are reported in Tables 2 and 3, showing the results of the comparative delta-Ct method, and the comparison with the NormFinder and geNorm results. Independently from the method used, the gene ranking remained unchanged, indicating Z30 and RNU24 as the best reference genes. In particular, the combination of Z30 and RNU24 was selected as the best among all the others, as underlined by the stability value of 0.011 calculated by NormFinder, the M value of 0.489 calculated by geNorm (see Additional file 1: Figure S1), and the delta-Ct values (Table 2). This combination showed the smallest and therefore best stability values among all the other combinations of reference genes (see Additional file 1: Figure S1) whereas the worst combination was attributed to RNU6B and miR-103a-3p.

**Table 2:** **Comparison of candidate reference genes expression stability using the comparative delta**-**Ct method**

Combination	Mean delta-Ct	SD	Mean SD
RNU24 vs Z30	-1.53	0.58	0.93
RNU24 vs RNU6B	-2.26	1.09	
RNU24 vs miR-103a-3p	-0.71	1.12	
Z30 vs RNU24	1.53	0.58	0.93
Z30 vs RNU6B	-0.73	0.99	
Z30 vs miR-103a-3p	0.82	1.23	
RNU6B vs Z30	0.73	0.99	1.25
RNU6B vs RNU24	2.26	1.09	
RNU6B vs miR-103a-3p	1.62	1.66	
miR-103a-3p vs Z30	-0.82	1.23	1.34
miR-103a-3p vs RNU24	0.71	1.12	
miR-103a-3p vs RNU6B	-1.62	1.66	

Mean delta-*Ct* = Ct target – Ct reference; *SD* = standard deviation of the mean; mean *SD* = mean of the calculated standard deviations.

**Table 3:** **Comparison of the stability values estimated using the comparative delta**-**Ct**, **NormFinder and GeNorm algoritms**

Gene	Comparative delta-Ct	NormFinder	GeNorm
RNU24	0.93	0.007	0.839
Z30	0.93	0.016	0.841
RNU6B	1.25	0.033	1.112
miR-103a-3p	1.34	0.018	1.310

Comparative delta-*Ct* = mean of the standard deviations (SD), as shown in Table 2; NormFinder = stability values computed with NormFinder and GeNorm: M stability values, calculated assuming the specific efficiency for each gene assay. NormFinder = stability values computed with NormFinder and GeNorm: M stability values, calculated assuming the specific efficiency for each gene assay.

Unexpectedly, miR-103a-3p, previously indicated as a suitable reference gene for quantification of microRNAs in peripheral blood of PD patients through qRT-PCR [8], was consistently found to be the worst reference gene in the analysed sample (Tables 2 and 3).

Furthermore, since the expression of reference genes is supposed to remain constant among different samples, we would expect that the relationship between the expression of two reference genes is linear.

To estimate the inter-gene relations of the four putative reference genes, correlation analysis was performed. Results indicate that the strength of the linear relationship is good for Z30 and RNU24 expression (r = 0.8004, p < 0.0001), which are those candidates ranked as the best reference genes. The correlation of the expression of RNU6B and miR-103a-3p with Z30 and RNU24 is smaller, with the lowest correlations observed between the expression levels of miR-103a-3p and the remaining reference genes (see Additional file 1: Table S1).

The same trend is also indicated by the scatter plot matrix (see Additional file 1: Figure S2), where the most linear relationship could be observed between Z30 and RNU24.

All the analyses were also repeated excluding outlier samples (sensitivity analyses), but no difference in the rank of the reference genes was observed (data not shown).

### Consequence of normalizing expression of miR-29a-3p and miR-30b-5p in PD cases and matched controls with different combinations of reference genes

We tested the effect of using different combinations of reference genes on the relative expression values using miR-29a-3p and miR-30b-5p as targets, which were found to be deregulated in PD in previous studies [7, 8].

The results of the Wilcoxon matched-pairs signed-ranks test (Table 4) show that there is an increased relative expression of miR-29a-3p and miR-30b-5p in patients with PD in comparison to controls.

**Table 4:** **Changes in the results of the analyses depending on the choice of the reference genes**

Reference gene	miR-29a-3p median of difference (IQR)	P value miR-29a-3p	miR-30b-5p median of difference (IQR)	P value miR-30b-5p
Z30	0.76 (-0.23; 2.22)	0.0048	1.75 (0.17; 3.62)	0.0007
RNU24	0.39 (-0.08; 1.34)	0.0151	0.96 (-0.52; 3.39)	0.0075
RNU6B	0.98 (-0.17; 2.12)	0.0007	2.66 (0.2; 4.05)	0.0001
miR-103a-3p	-0.58 (-1.33; -0.05)	0.0003	-1.16 (-2.53; 0.23)	0.0005
Z30- miR-103a-3p	-0.02 (-0.65; 0.6)	0.7997	0.01 (-1.14; 1.32)	0.8221
Z30-RNU24*	0.68 (-0.17; 1.74)	0.0032	1.74 (0.05; 4.26)	0.0009
Z30-RNU24- miR-103a-3p	0.18 (-0.48; 0.71)	0.5473	0.22 (-1.1; 1.63)	0.3422
Z30-RNU24-RNU6B- miR-103a-3p	0.38 (-0.31; 1.23)	0.0511	1.05 (-0.31; 2.41)	0.0092
RNU24-RNU6B	0.85 (-0.02; 1.77)	0.0011	2.37 (0.2; 3.47)	0.0004
RNU24- miR-103a-3p	-0.35 (-0.75; 0.15)	0.0645	-0.7 (-1.54; 1.13)	0.2372
RNU24-RNU6B- miR-103a-3p	0.12 (-0.31; 0.91)	0.2097	0.61 (-0.44; 1.95)	0.0494
RNU24-RNU6B-Z30	0.74 (-0.08; 1.86)	0.0013	2.02 (0.1; 3.95)	0.0003
RNU6B- miR-103a-3p	0.04 (-0.41; 0.66)	0.3962	0.51 (-0.4; 1.19)	0.0939
RNU6B-Z30	0.79 (-0.1; 1.98)	0.0011	2.44 (0.17; 3.8)	0.0003
RNU6B-Z30- miR-103a-3p	0.31 (-0.36; 1.01)	0.0733	1.02 (-0.3; 1.99)	0.0119

Different combinations of reference genes lead to different relative expression values of miR-29a-3p and miR-30b-5 in the matched PD pairs, therefore affecting the results of the statistical analyses and leading to discordant evidence. *Best set of reference genes.

The median of difference is calculated as median (expression in cases – expression in controls) within each matched case–control set and it is indicated for a descriptive purpose. IQR = interquartile range. P values were computed using a Wilcoxon matched-pairs signed-ranks test.

Normalizing the expression values of miR-29a-3p and miR-30b-5p for the best reference gene combination of Z30 and RNU24 results in a median difference of the relative expression of 0.68 (IQR = -0.17; 1.74) for miR-29a-3p (p value = 0.0032), and equal to 1.74 (IQR = 0.05; 4.26) for miR-30b-5p (p value =0.0009). This trend was consistently observed using Z30, RNU24 and RNU6B as reference genes, either alone or in different combinations (Table 4).

However, the normalization of the microRNA expression using miR-103a-3p as reference gene results in an opposite effect, with a lower expression of miR-29a-3p and miR-30b-5p in cases, in comparison with controls. The usage of miR-103a-3p alone as a reference gene was sufficient to completely reverse the direction of the expression difference between cases and controls of the investigated miRNAs (miR-29a-3p median difference = -0.58, IQR = -1.33; -0.05, p = 0.0003 and miR-30b-5p median difference = -1.16, IQR = -2.53; 0.23, p = 0.0005).

The inclusion of miR-103a-3p in any other combination of reference genes systematically worsened the observed p values. This effect was clearly visible with the addition of miR-103a-3p to the combination of Z30 and RNU24, which had a strong impact on the results observed for both miR-29a-3p and miR-30b-5p, with the p value of miR-29a-3p changing from p = 0.0032 to p = 0.5473 and the p value of miR-30b-5p changing from p = 0.0009 to p = 0.3422. Indeed, the relative expression levels of miR-29a-3p/miR-30b-5p were not always well correlated with each other. But some differences could be observed, depending on the reference gene set used for normalization, as shown in Additional file 1: Tables S2 and S3. Smaller correlation coefficients could be observed when miR-103a-3p alone was used as a reference gene, while correlations were improved when it was present in other combinations.

Sensitivity analyses were finally repeated excluding outlier samples, but no difference in the results was observed (data not shown).

## Discussion

Quantitative real-time PCR is the most commonly available and reliable method used in expression profile studies aimed at detecting changes in gene expression, because alternative approaches do not yet match the sensitivity and specificity of PCR-based approach. The choice of housekeeping genes in a qRT-PCR study is a critical step before starting any experiment, in order to avoid inaccurate interpretation of the data that may lead to biased results. Indeed, it has been shown in several publications that traditional reference genes used in qRT-PCR studies do not always show a stable expression pattern. The same gene revealed as almost invariant for certain tissues or cell types, could present highly variable expression levels in other tissues or experimental conditions [20, 25, 26]. Thus, suitable control genes are extremely specific for particular sample sets and experimental models, being a crucial component in assessing gene expression patterns with confidence. Striking examples are provided by the glyceraldehyde-3-phosphate dehydrogenase (*GAPDH*) and beta-actin genes, which have been extensively used for normalization of gene expression data in a broad range of different tissues and pathologies, including the nervous system [25–28]. It has been shown however, that *GAPDH* and beta-actin genes are indeed direct targets of miR-644a in prostate cancer cell lines, demonstrating the unsuitability of *GAPDH* and beta-actin as internal controls in miR-644a functional studies and emphasizing the need to carefully consider the choice of a reference gene according to the specific study design [29].

Small nuclear RNAs as well as miRNAs and snoRNAs are widely used as reference genes for miRNA studies in tissue samples and blood. To date, in the field of the Parkinson's disease there is no work reporting the validation of a set of reliable reference genes for a miRNA profiling study in blood PD samples.

For that reason, the present study aims not only to identify and to validate specific endogenous reference genes as reported in the literature, but also to evaluate the impact of the use of different combinations of these reference genes on the interpretation of expression profile differences between cases and controls of certain target miRNAs.

We selected from the literature the most commonly used reference genes in qRT-PCR blood studies or in neurological diseases studies. We then assessed their suitability as normalizers, and their effect, alone or in combination, on the target genes miR-30b-5p and miR-29a-3p in PD blood samples, previously shown to have altered expression profiles in PD [7, 8]. The selection of the best set of endogenous reference genes for gene expression studies in Parkinson’s disease blood samples from those tested was based on the efficiency of the TaqMan® MicroRNA Assays, the quality of the related expression data, on the expression stability analysis, and biological data available in the literature.

Using these metrics of efficiency, quality and stability, RNU24 ranks top of the list, followed by Z30 as the best reference genes in our study. Moreover, neither RNU24 nor Z30 have been related so far to neurodegeneration and/or Parkinson’s disease. On the contrary, miR-103a-3p is ranked as the worst reference gene, so much so that in combination with other reference genes it is able to bias results.

It is important to underline that miR-103a-3p alone or in combination with the other reference genes reverses the direction of the expression of miR-29a-3p and miR-30b-5p. Our results are consistent with the available literature evidence, which support the unsuitability of miR-103a-3p as a reference gene in PD studies for a number of reasons. MiR-103a-3p and miR-107 have been recently shown to target human *CDK5R1* [30], coding for the regulatory subunit 1 of cyclin-dependent kinase 5, a protein found to be hyperactivated in different neurodegenerative diseases, including Parkinson’s and Alzheimer's diseases [31, 32]. The same two miRNAs have also been shown to regulate insulin sensitivity by targeting caveolin-1 [33]. The connection between miR-103a-3p and insulin is relevant, since insulin is known to regulate dopamine release. Moreover, in a recent study a correlation between PD and a hyperglycaemic status was shown in rats [34]. Last, but not of minor importance when studying neurodegenerative disease, is the evidence that the expression of miR-103a-3p varies depending on the age of the patients [35]. In our study, the paired case–control experimental design allows correction for the confounding effect of age, but adjustment for age would be required for unmatched designs. This may be unfeasible for small qRT-PCR studies, therefore the confounding effect would not be controlled and the results would be biased.

Contrary to miR-103a-3p, the insertion of RNU6B in combinations with other reference genes has a lower impact on the stability values, but we hypothesize that this effect can be attributed to the high stability of RNU24 and Z30. RNU6B was not considered to be a reliable reference gene for PD blood samples in our study, because the efficiency, the r^2^ and the stability values were too low. Furthermore, in support of this conclusion, RNU6B has been shown to be stable under some but not all conditions of neuronal differentiation [36].

## Conclusion

In conclusion, the results of this study indicate that relative expression differences of the selected microRNAs between Parkinson’s cases and controls were profoundly affected by the choice of the reference gene set, thus demonstrating the complexity of the choice of reference gene set. A real endogenous reference gene should be stably expressed, regardless of physiological conditions, including age and disease. In our hands, using miR-103a-3p as a reference gene, alone or in combination with other suitable genes, produced contrasting results within our dataset. Our data suggest that RNU24 and Z30 constitute a reference gene set that will lead to reliable data for normalization of miRNA expression profiles in blood of PD patients versus controls, at least until new technologies for direct PCR quantification such as droplet-digital PCR become more widespread and affordable. The results confirm the importance of a careful choice of the reference genes, which should be evaluated depending on the specific on-going study, not relying solely on evidence already published.

## Electronic supplementary material

Additional file 1: **The file contains the additional tables S1-S3 and the additional figures S1-S2.** (PDF 110 KB)

## Abbreviations

miRNAs: microRNAs
PD: Parkinson’s disease
RT-qPCR: Reverse transcription and quantitative real time polymerase chain reaction
RT: Reverse Transcription
PBMCs: Peripheral blood mononucleated cells
RQI: RNA quality indicator
Ct: Threshold cycle.
