# Supplementary material for: Identification of a set of endogenous reference genes for miRNA expression studies in Parkinson’s disease blood samples
Source: BMC Res Notes. 2014 Oct 10;7:715. doi: 10.1186/1756-0500-7-715 (PMC4209045; doi:10.1186/1756-0500-7-715)
Supplement: Supplementary file 1 — Additional file 1: The file contains the additional tables S1-S3 and the additional figures S1-S2. (PDF 110 KB) [file 13104_2014_3248_MOESM1_ESM.pdf]

SUPPLEMENTARY TABLES AND FIGURES

Table S1. Correlation matrix of reference genes expression. Spearman correlation coefficients were calculated; p values are reported in parenthesis.

	**Z30**	**RNU24**	**RNU6B**	**miR-103a-3p**
**Z30**	1			
**RNU24**	0.8004 (<0.0001)	1		
**RNU6B**	0.1221 (0.0528)	0.2504 (0.0001)	1	
**miR-103a-3p**	0.6619 (<0.0001)	0.6520 (<0.0001)	0.0179 (0.7780)	1

Figure S1. M stability values of gene combinations. Dot plot showing the M stability values computed for each combination of the analysed reference genes using geNorm. miR103 is an abbreviation for miR-103a-3p.

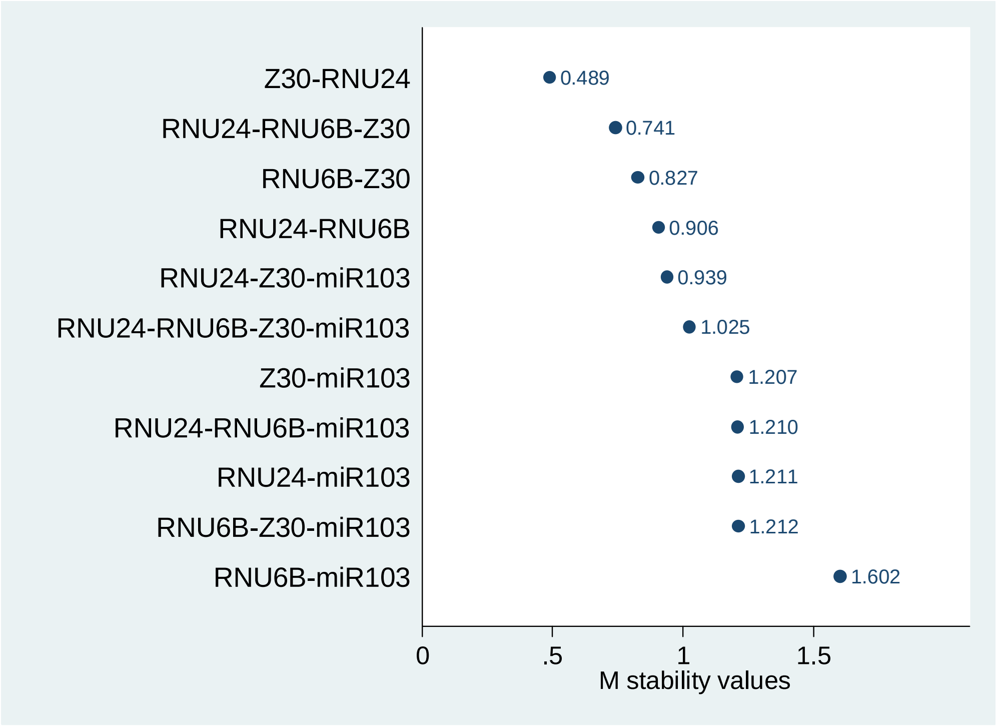

Figure S2. Scatter plots of the expression levels in candidate reference genes. Data plotted are Ct values of the reference genes. miR103 is an abbreviation for miR-103a-3p.

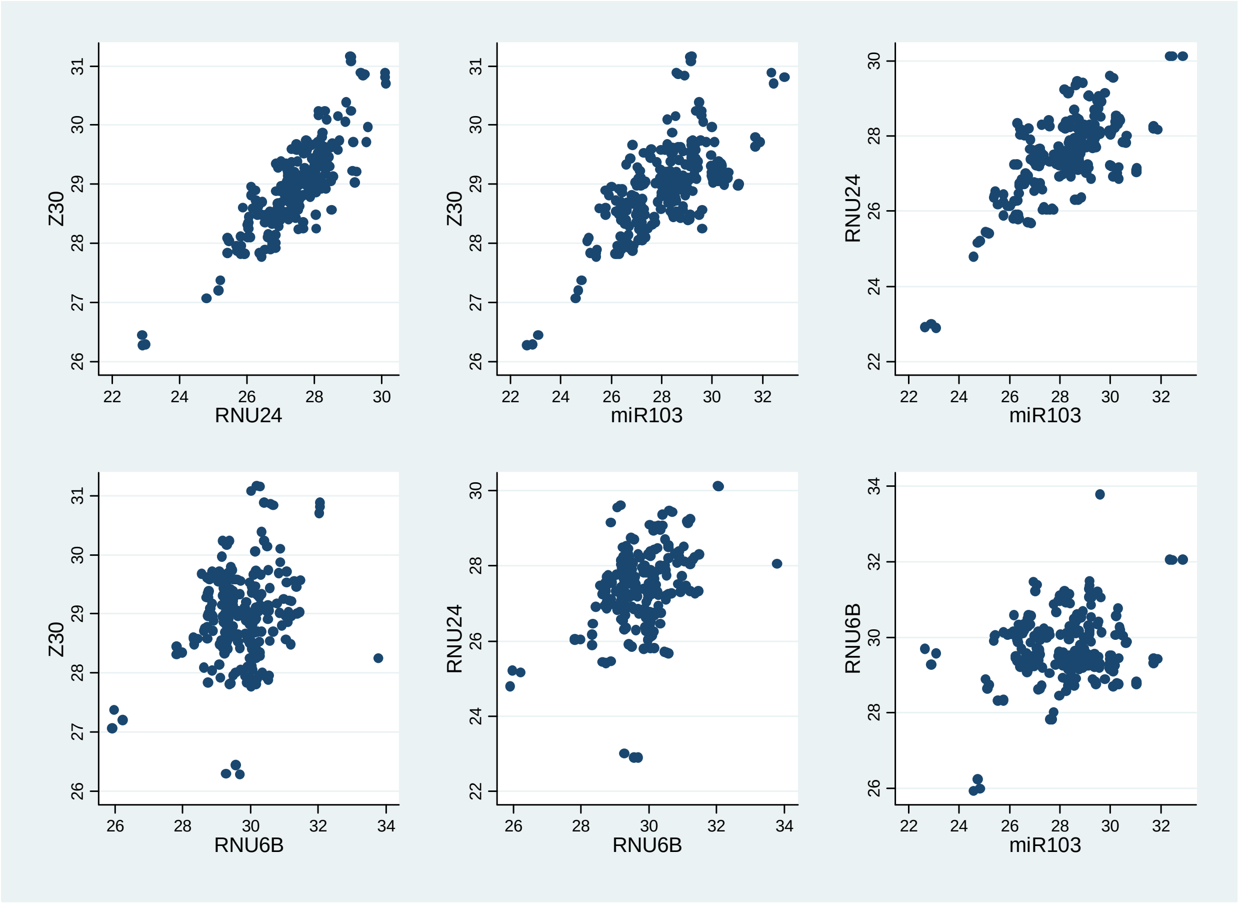

Table S2. Correlation of miR29a relative expression levels using different reference genes. Spearman correlation coefficients were computed. All p values are <0.001. Abbreviations: miR103 = miR-103a-3p, miR-29a = miR-29a-3p.

TARGET: miR-29a	Z30	RNU24	RNU6B	miR103	RNU24-RNU6B	RNU24-miR103	RNU24-RNU6B-miR103	RNU24-RNU6B-Z30	RNU24-Z30-miR103	RNU24-RNU6B-miR103	RNU6B-Z30	RNU6B-Z30-miR103	Z30-miR103	Z30-RNU24	ALL
**Z30**	1														
**RNU24**	0.8452	1													
**RNU6B**	0.8209	0.7773	1												
**miR103**	0.3882	0.4237	0.3207	1											
**RNU24-RNU6B**	0.88	0.9031	0.9612	0.3808	1										
**RNU24-miR103**	0.6717	0.7757	0.5927	0.8763	0.6994	1									
**RNU24-RNU6B-miR103**	0.8412	0.8706	0.8796	0.643	0.936	0.8755	1								
**RNU24-RNU6B-Z30**	0.9435	0.9065	0.9399	0.3857	0.9849	0.7031	0.9269	1							
**RNU24-Z30-miR103**	0.8838	0.8774	0.7437	0.7113	0.8445	0.9249	0.9408	0.8778	1						
**RNU24-RNU6B-miR103**	0.7637	0.7423	0.844	0.7268	0.858	0.873	0.9664	0.8469	0.8959	1					
**RNU6B-Z30**	0.9374	0.8444	0.9626	0.3611	0.9695	0.6563	0.9039	0.987	0.8398	0.8477	1				
**RNU6B-Z30-miR103**	0.9034	0.8367	0.8919	0.6053	0.9251	0.8282	0.9716	0.944	0.9411	0.9513	0.9402	1			
**Z30-miR103**	0.8154	0.7336	0.647	0.8173	0.7275	0.9272	0.8815	0.7734	0.9623	0.892	0.752	0.8994	1		
**Z30-RNU24**	0.971	0.939	0.8365	0.4052	0.9288	0.7335	0.8905	0.9686	0.9145	0.7864	0.9365	0.9121	0.8123	1	
**ALL**	0.9158	0.8984	0.8872	0.5784	0.949	0.8404	0.9808	0.9634	0.9581	0.9288	0.9421	0.9865	0.8892	0.9471	1

Table S3. Correlation of miR30b relative expression levels using different reference genes. Spearman correlation coefficients were computed. Some p values are specified in parenthesis, all remaining p values are <0.001. Abbreviations: miR103 = miR-103a-3p, miR-30b = miR-30b-5p.

TARGET: miR-30b	Z30	RNU24	RNU6B	miR103	RNU24-RNU6B	RNU24-miR103	RNU24-RNU6B-miR103	RNU24-RNU6B-Z30	RNU24-Z30-miR103	RNU24-RNU6B-miR103	RNU6B-Z30	RNU6B-Z30-miR103	Z30-miR103	Z30-RNU24	ALL
**Z30**	1														
**RNU24**	0.8057	1													
**RNU6B**	0.7946	0.7219	1												
**miR103**	0.242 (0.0328)	0.331 (0.0031)	0.1711 (0.1341)	1											
**RNU24-RNU6B**	0.8706	0.8774	0.9588	0.2301 (0.0427)	1										
**RNU24-miR103**	0.8051	0.8366	0.8451	0.5731	0.9036	1									
**RNU24-RNU6B-miR103**	0.591	0.7328	0.4793	0.8487	0.6006	0.8431	1								
**RNU24-RNU6B-Z30**	0.9301	0.8854	0.93	0.2307	0.9864	0.8931	0.6094	1							
**RNU24-Z30-miR103**	0.8224	0.8304	0.6594	0.6707	0.7757	0.9296	0.9275	0.8078	1						
**RNU24-RNU6B-miR103**	0.7269	0.6703	0.8193	0.6306	0.8194	0.9595	0.8125	0.8024	0.8787	1					
**RNU6B-Z30**	0.9317	0.8008	0.9527	0.2073	0.9688	0.8695	0.5551	0.9837	0.7699	0.8152	1				
**RNU6B-Z30-miR103**	0.8734	0.7661	0.8573	0.5304	0.8879	0.9671	0.7895	0.9016	0.9227	0.9634	0.9105	1			
**Z30-miR103**	0.7645	0.6856	0.5763	0.7559	0.6638	0.8744	0.9199	0.7042	0.9677	0.8752	0.6936	0.8984	1		
**Z30-RNU24**	0.9509	0.9366	0.8009	0.2987	0.9211	0.8679	0.688	0.9574	0.8737	0.7421	0.9149	0.8692	0.7693	1	
**ALL**	0.8907	0.8588	0.868	0.4891	0.932	0.9815	0.7963	0.9433	0.9331	0.9293	0.9258	0.9814	0.8732	0.9259	1
